# Retraction: Indomethacin-based stimuli-responsive micelles combined with paclitaxel to overcome multidrug resistance

**DOI:** 10.18632/oncotarget.28753

**Published:** 2025-07-09

**Authors:** Shuanghu Wang, Xueying Tan, Shujuan Li, Yunfang Zhou, Peiwu Geng, Ailian Hua, Aiping Deng, Zhihong Yu

**Affiliations:** ^1^The Laboratory of Clinical Pharmacy, The Sixth Affiliated Hospital of Wenzhou Medical University, The People’s Hospital of Lishui, Lishui 323000, China; ^2^College of Pharmacy, Zhejiang Pharmaceutical College, Ningbo 315000, China; ^3^Department of Pharmacy, The Central Hospital of Wuhan, Tongji Medical College, Huazhong University of Science and Technology, Wuhan 430000, China; ^*^These authors have contributed equally to this work

**This article has been retracted:** This decision follows an investigation by Oncotarget journal regarding the concerns raised by the third party. Our image forensics analysis revealed several issues:

- In Figure 3B, the fluorescence image of cells treated with DEX-IND/NR for 10 hours is a duplicate of an image in Figure 4A from a 2017 paper with common authors [1], where it is described differently.
- In Figure 6C, the MCF-7/PTX xenograft tumor sections stained with TUNEL have an overlapping image in the Saline group with the TUNEL/Saline image in Figure 7C of the same 2017 paper [1].
- The panel representing H@E staining of the liver in the DEX-SS-IND treatment group in Figure 7C was later reused as the Saline liver treatment in Figure 6E of a 2022 paper with common authors [2], which has since been retracted.

Furthermore, recently, after investigation, review and approval, the National Natural Science Foundation of China (NNSFC) concluded that this Oncotarget article contained image forgery and tampering, as well as the unauthorized use of other people’s names. Corresponding author Yu Zhihong has been identified as responsible for these issues.

Considering both the NNSFC findings and the results of our journal investigation, the editorial decision has been made to retract the article. All authors were informed of the retraction, but either did not respond directly or could not be reached.

Original article: Oncotarget. 2017; 8:111281–111294. 111281-111294. https://doi.org/10.18632/oncotarget.22781
